# Preparation of Amylose-Oligo[(*R*)-3-hydroxybutyrate] Inclusion Complex by Vine-Twining Polymerization

**DOI:** 10.3390/molecules26092595

**Published:** 2021-04-29

**Authors:** Jun-ichi Kadokawa, Yuki Wada, Kazuya Yamamoto

**Affiliations:** Graduate School of Science and Engineering, Kagoshima University, 1-21-40 Korimoto, Kagoshima 890-0065, Japan; k9350478@kadai.jp (Y.W.); yamamoto@eng.kagoshima-u.ac.jp (K.Y.)

**Keywords:** amylose, enzymatic polymerization, glucan phosphorylase, inclusion complex, oligo[(*R*)-3-hydroxybutyrate], vine-twining polymerization

## Abstract

In this study, we attempted to prepare an amylose-oligo[(*R*)-3-hydroxybutyrate] (ORHB) inclusion complex using a vine-twining polymerization approach. Our previous studies indicated that glucan phosphorylase (GP)-catalyzed enzymatic polymerization in the presence of appropriate hydrophobic guest polymers produces the corresponding amylose–polymer inclusion complexes, a process named vine-twining polymerization. When vine-twining polymerization was conducted in the presence of ORHB under general enzymatic polymerization conditions (45 °C), the enzymatically produced amylose did not undergo complexation with ORHB. However, using a maltotriose primer in the same polymerization system at 70 °C for 48 h to obtain water-soluble amylose, called single amylose, followed by cooling the system over 7 h to 45 °C, successfully induced the formation of the inclusion complex. Furthermore, enzymatic polymerization initiated from a longer primer under the same conditions induced the partial formation of the inclusion complex. The structures of the different products were analyzed by X-ray diffraction, ^1^H-NMR, and IR measurements. The mechanism of formation of the inclusion complexes discussed in the study is proposed based on the additional experimental results.

## 1. Introduction

Amylose is an abundant polysaccharide composed of α(1→4)-linked glucose (G) repeating units [1]. Besides its role in nature as an energy resource as a component of starch, amylose has been identified as a functional supramolecular material owing to its left-handed helical conformation with controlled regularity [2,3]. For example, as the inside of the helix is hydrophobic, amylose readily complexes within its cavity hydrophobic guest molecules of appropriate sizes and geometries, such as fatty acids, to form supramolecular inclusion complexes [4].

The complete separation of amylose from amylopectin, the other component in natural starch sources, is difficult. However, an enzymatic approach using α-glucan phosphorylase (GP) catalysis to form a regio- and stereo-controlled α(1→4)-glycosidic arrangement is a powerful tool to obtain pure amylose [5,6,7,8,9,10]. GP catalyzes the enzymatic polymerization of α-d-glucose 1-phosphate (G-1-P), the monomer, initiated from a maltooligosaccharide primer, resulting in the formation of α(1→4)-glucan (amylose) according to the following reversible reaction: [α(1→4)-G]*_n_* + G-1-P ⇄ [α(1→4)-G]*_n+1_* + inorganic phosphate. GP-catalyzed enzymatic polymerization under ambient conditions, such as at 45 °C, yields an insoluble amylosic product in aqueous media through the formation of a well-known double-helical assembly [11,12]. We found that enzymatic polymerization using thermostable GP (isolated from the thermophilic bacterium *Aquifex aeolicus* VF5) at elevated temperatures, for example, at 70 °C, produced a water-soluble amylosic product with a single chain (called single amylose) in an equilibrium state between chain elongation and cleavage reactions (polymerization and phosphorolysis, respectively) [13].

Interestingly, when the GP-catalyzed enzymatic polymerization is conducted in the presence of appropriate hydrophobic polymers dispersed in aqueous buffer solvents, these polymers are gradually included in the helical cavity of the produced amylose chains during the propagation from the short primer toward longer α(1→4)-glucan chains, leading to the formation of amylose–polymer inclusion complexes [14,15,16,17,18]. As a controlled water-insoluble assembly is formed, these complexes gradually precipitate in the system. As the polymerization resembles the growth of plant vines that gradually twine around a support rod, the system was termed vine-twining polymerization. Both hydrophilic and strongly hydrophobic polymers are not suitable as guests for vine-twining polymerization as they undergo weak hydrophobic interactions with the amylosic cavity and aggregate in aqueous media, respectively [19,20]. Therefore, polyesters with moderate hydrophobicity, such as poly(ε-caprolactone) (PCL), poly(δ-valerolactone) (PVL), and poly(l-lactide), are ideal guests for vine-twining polymerization and have been shown to form the corresponding amylosic inclusion complexes [21,22,23].

**Scheme 1:**
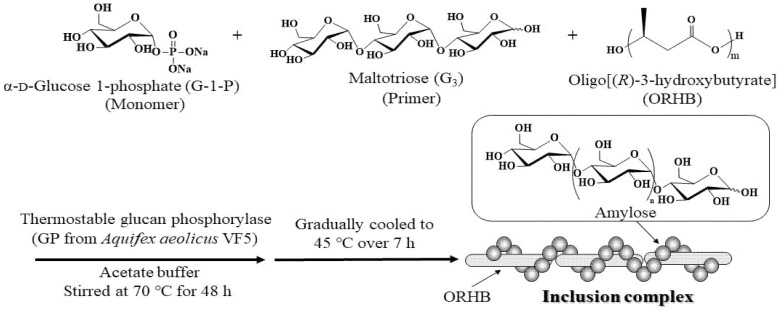
Preparation of amylose–ORHB inclusion complex by vine-twining polymerization under conditions at controlled temperatures.

We attempted to use poly[(*R*)-3-hydroxybutyrate] (PRHB), a well-known polyester biosynthesized by microorganisms [24], as a new guest polyester with a similar structure to those discussed above for vine-twining polymerization. Owing to the high molecular weight (MW = ~500,000) and strong hydrophobicity of biosynthesized PRHB, our attempt at vine-twining polymerization under typical conditions at 45–50 °C mostly has not induced the inclusion of PRHB into the cavity of amylose. Therefore, in this study, we employed low-molecular-weight oligo[(*R*)-3-hydroxybutyrate] (ORHB) (<1000), prepared by partial alkaline hydrolysis of the parent PRHB, as the guest for the vine-twining polymerization. Thermostable GP-catalyzed enzymatic polymerization in the presence of ORHB was found to produce the amylose–ORHB inclusion complexes under controlled temperature conditions, as shown in Scheme 1. In addition, this study focuses on the extension of guest polymers, i.e., ORHB, in the vine-twining polymerization and, furthermore, the amylose–ORHB inclusion complex can be expected as a new bio-based and biocompatible material owing to biodegradability in both the components.

## 2. Results and Discussion

**Figure 1:**
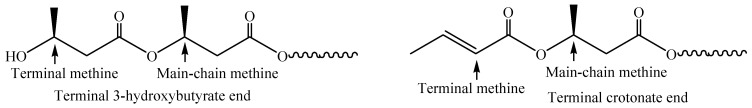
Terminal structures of ORHB.

The low-molecular-weight ORHB was first prepared by partial alkaline hydrolysis of a commercially available PRHB (MW = ~500,000) in the presence of sodium hydroxide in chloroform at room temperature for 7 h. The presence of signals corresponding to both terminal 3-hydroxybutyrate and crotonate (derived by the dehydration of terminal 3-hydroxybuturate) in the ^1^H-NMR spectrum of the isolated product in CDCl_3_ supported the formation of the desired ORHB (Figure 1) [25]. Based on the ratio of the integrals of the terminal methine signals to the main-chain methine signals, the MW of the product was calculated to be, e.g., ~530.

**Figure 2:**
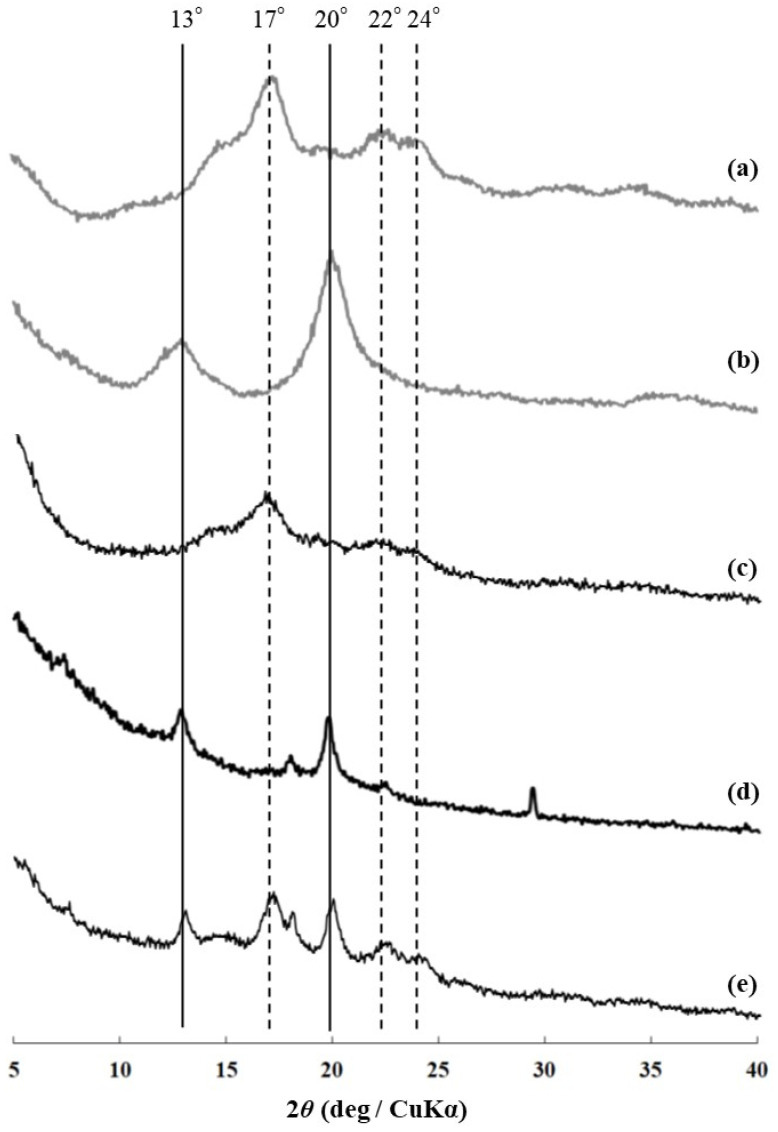
XRD profiles of (**a**) amylose, (**b**) amylose–PCL inclusion complex, (**c**) product obtained at 45 °C, (**d**) product obtained using G_3_ primer at elevated temperature, and (**e**) product obtained using water-soluble amylose primer at elevated temperature.

**Figure 3:**
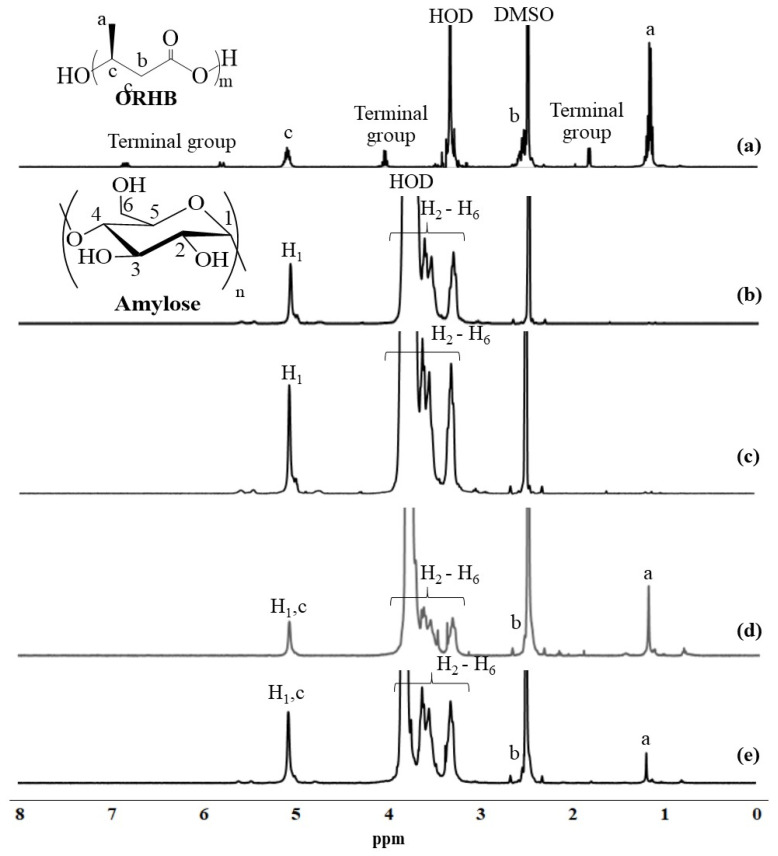
^1^H-NMR spectra of (**a**) ORHB in DMSO-*d*_6_, (**b**) amylose, (**c**) product obtained at 45 °C, (**d**) product obtained using G_3_ primer at elevated temperature, and (**e**) product obtained using water-soluble amylose primer at elevated temperature in DMSO-*d*_6_ + D_2_O.

**Figure 4:**
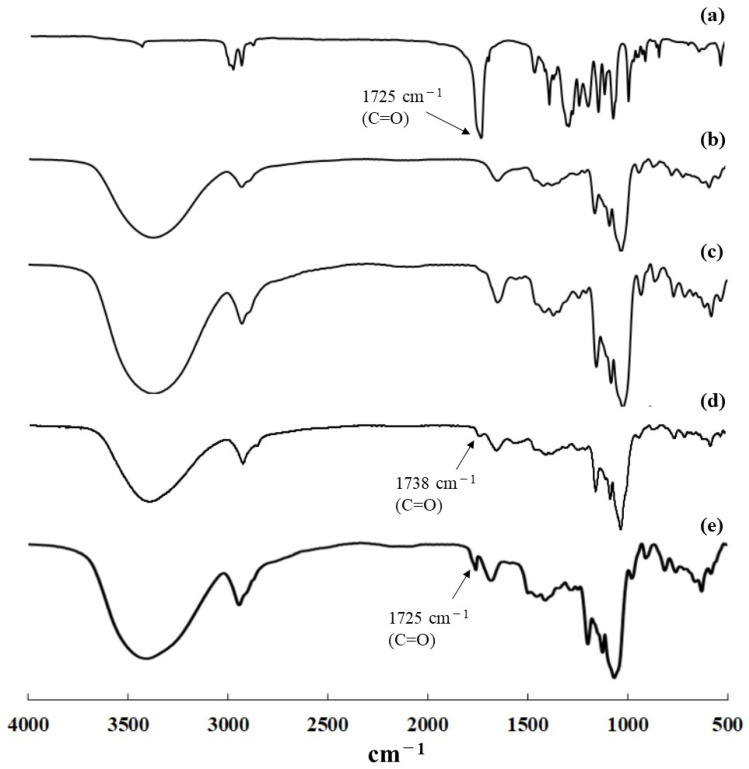
IR spectra of (**a**) ORHB, (**b**) amylose, (**c**) product obtained at 45 °C, (**d**) product obtained using G_3_ primer at elevated temperature, and (**e**) product obtained using water-soluble amylose primer at elevated temperature.

Vine-twining polymerization using ORHB was first performed by the thermostable GP (from *Aquifex aeolicus* VF5)-catalyzed enzymatic polymerization of G-1-P from a maltoheptaose primer in acetate buffer solvent under the typical conditions reported in our previous studies: ORHB was dispersed in the mixture at 45 °C for 24 h with vigorous stirring [21,22]. The precipitate was isolated by centrifugation, washed successively with water, acetone, and chloroform, and subsequently dried under reduced pressure to obtain the product. The powder X-ray diffraction (XRD) profile of the product was identical to that of the pure double-helical amylose (17°, 22°, and 24°), but completely different from that of the previously reported amylose–PCL inclusion complex (13° and 20°) [21,22] (Figure 2a–c). The ^1^H-NMR in DMSO-*d*_6_ + D_2_O and IR spectra of the product showed signals corresponding to pure amylose but did not exhibit characteristic signals derived from ORHB, such as a methyl signal and a carbonyl absorption peak, respectively (Figure 3a–c and Figure 4a–c). These results strongly suggest that under these conditions, the enzymatically produced amylose did not include ORHB, and instead favorably formed a double helix structure.

We then attempted to obtain the inclusion complex from ORHB by enzymatic production through the formation of water-soluble amylose without a double-helical assembly, called single amylose, at elevated temperatures (Scheme 1) [13]. The thermostable GP-catalyzed enzymatic polymerization of G-1-P from a maltotriose (G_3_) primer (80:1) was conducted in the presence of ORHB (MW = 530) dispersed in an acetate buffer solvent. The mixture was maintained at 70 °C for 48 h; we have already confirmed that such elevated reaction temperatures in the thermostable GP-catalyzed enzymatic polymerization do not affect the chemical structure of the produced amylose [13,23]. Subsequently, to permit the further progress of the polymerization and simultaneously accelerate the complexation and precipitation, the mixture was stirred and gradually cooled to a temperature of 45 °C over 7 h. The product was then isolated using the same procedure as described above and characterized by XRD, ^1^H-NMR, and IR measurements. The XRD profile of the isolated product showed diffraction peaks at 13° and 20°, which was identical to that of the 6_1_ helix in the amylose–PCL inclusion complex [26], and completely different from that of the pure amylose (Figure 2a,b,d), suggesting the successful formation of the inclusion complex. The structure of the inclusion complex was also supported by the ^1^H-NMR and IR results. The ^1^H-NMR spectrum of the isolated product in DMSO-*d*_6_ + D_2_O showed signals corresponding to both amylose and ORHB (Figure 3d). Generally, one helical turn of amylose is composed of approximately six repeating G units when linear molecules, such as PCL, are included, as suggested by the corresponding XRD profiles (Figure 2b,d) that show a repeat distance of 0.800 nm for the amylose helix [27]. On the other hand, the unit length of ORHB was calculated to be 0.446 nm. Therefore, an average of 3.38 repeating G units in amylose corresponds to the length of one unit of ORHB. Based on these calculations, the ratio of the integrals of the H_1_ (anomeric) signals at δ 4.98–5.18 to the methyl signal **a** at δ 1.19 (**a**/H_1_) in the ^1^H NMR spectrum is theoretically 0.897. The **a**/H_1_ value in the ^1^H NMR spectrum of the product in Figure 3d was calculated to be 1.18, which is slightly higher than the theoretical value. This indicates that the amylose cavity quantitatively includes ORHB. The IR spectrum of the product exhibited a band corresponding to ester C=O absorption at 1738 cm^−1^ (Figure 4d). This band indicated the presence of ORHB, although it was detected at a different wavenumber from the carbonyl absorption of pure crystalline ORHB, at 1725 cm^−1^ (Figure 4a), similar to the absorption shift reported in a previous study on vine-twining polymerization using PVL [22]. The considerable difference in these peaks is because of the non-crystalline nature of ORHB in the product, indicating that the inclusion of amylose prevents the formation of a crystalline structure in the product. Dissociation of the inclusion complex was carried out by the treatment with DMSO at elevated temperatures according to our reported procedure [28]. The degree of polymerization (DP) and MW of the dissociated amylose were determined from the *λ*_max_ value in the UV–Vis spectra of the violet solution of a complex with iodine to be 62.3 and 10,100, respectively [29,30]. The lengths of the amylose helix and the ORHB chain estimated from their DP values (62.3 and 3.95, respectively) were 8.3 and 1.76 nm, respectively, which suggests that one amylose chain includes, on average, four or five ORHB molecules. From the amylose/ORHB unit ratio of 1:0.28 in the product (calculated by the actual **a**/H_1_ value = 1.18), which corresponded to their weight ratio of 1:0.15, the yield of the inclusion complex was calculated to be ca. 10% based on the amounts of the G_3_ primer in the feed and the product (5.5 and 12.6 mg, respectively, Section 3.3). The relatively low yield of the amylosic inclusion complex owed to equilibrium nature of the GP-catalyzed enzymatic polymerization, as a similar value was reported in the vine-twining polymerization using PCL [21].

**Figure 5:**
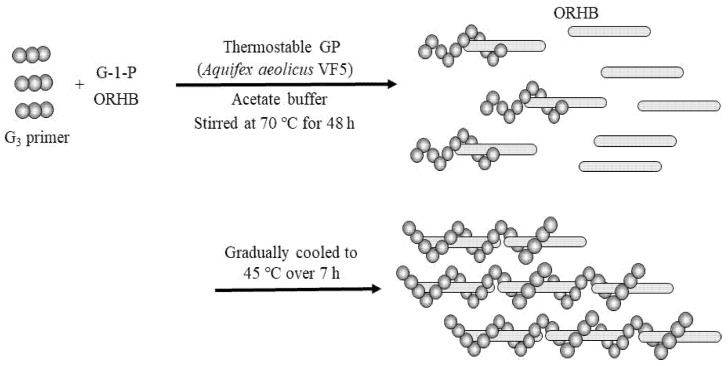
Plausible process for formation of amylose–ORHB inclusion complexes in thermostable GP-catalyzed enzymatic polymerization under conditions at controlled temperatures.

The following process is proposed for the formation of the amylose–ORHB inclusion complex during the enzymatic polymerization (Figure 5). At an elevated temperature (70 °C), the thermostable GP-catalyzed enzymatic polymerization from the G_3_ primer produces water-soluble single amylose with some chain elongation. To reach equilibrium during chain elongation, the single amylose weakly interacts with ORHB. The cooling of the reaction mixture to 45 °C over 7 h induces the weak assembly of complexes, shifting the equilibrium to further chain elongation. The elongated amylose chain then includes additional ORHB molecules to form a water-insoluble assembly from the regular inclusion complexes.

**Figure 6:**
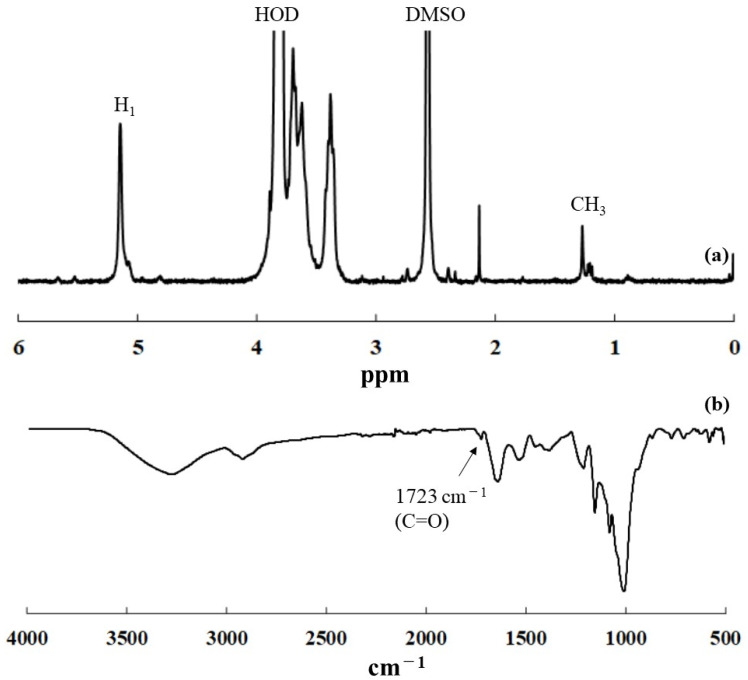
(**a**) ^1^H-NMR spectrum in DMSO-*d*_6_ + D_2_O and (**b**) IR spectrum of the interaction product between single amylose and ORHB.

To support the above hypothesis, thermostable GP-catalyzed enzymatic polymerization was conducted in the presence of ORHB dispersed in acetate buffer solvent at 70 °C for 48 h to attain equilibrium with the production of single amylose. The resulting reaction mixture was immediately subjected to centrifugation without cooling to isolate a water-insoluble fraction. The resulting product was washed with water and chloroform. Despite thorough washing with appropriate solvents to remove the single amylose (water) and ORHB (chloroform), the ^1^H-NMR spectrum of the product in DMSO-*d*_6_ + D_2_O showed a methyl signal at δ 1.27 from ORHB and an H_1_ signal at δ 5.15 from amylose (Figure 6a). In addition, the IR spectrum of the product exhibited a carbonyl absorption at 1723 cm^−1^ (Figure 6b), indicating the presence of ORHB in the product. These analytical results indicated the occurrence of some interaction between the single amylose and ORHB, which may be a key material at the initial stage for the formation of the inclusion complex in the present system.

To confirm the necessity of the chain-elongation process from the shorter G_3_ to the longer single amylose at the early stage of complexation, thermostable GP-catalyzed enzymatic polymerization was performed in the presence of a water-soluble amylose (MW = 2800) as the primer instead of G_3_. The reaction was performed under similar conditions as above, at controlled temperatures (70 °C for 48 h and subsequent cooling to 45 °C over 7 h). The XRD profile of the product obtained using the same isolation procedure showed diffraction peaks that could be assigned to both the amylosic inclusion complex at 13° and 20° and the double-helical amylose at 17°, 22°, and 24° (Figure 2e). This result indicated that the enzymatically produced amylose from the water-soluble amylose primer partially formed an inclusion complex with ORHB. Although the ^1^H-NMR spectrum of the product in DMSO-*d*_6_ + D_2_O showed a methyl signal at δ 1.26 from ORHB (Figure 3e), its carbonyl absorption in the IR spectrum was detected at 1725 cm^−1^, which corresponded to the crystalline structure (Figure 4e). The IR data suggested that ORHB was partly included in the cavity of amylose, and the excluded ORHB chains probably formed a crystalline structure. However, the formation of the crystalline structure from the ORHB in the product was not confirmed by XRD measurements, because the diffraction peaks ascribed to the ORHB crystal completely overlapped with the peaks from the inclusion complex and the double helix of amylose (data not shown).

## 3. Materials and Methods

### 3.1. Materials

Thermostable GP from *Aquifex aeolicus* VF5 was supplied by Ezaki Glico Co., Ltd. (Osaka, Japan) [31,32]. An amylose sample was prepared by the thermostable GP-catalyzed enzymatic polymerization of Glc-1-P [9]. The amylose–PCL inclusion complex was prepared according to a procedure from the literature [21]. The biosynthesized PRHB with MW = ~500,000 and water-soluble amylose with MW = 2800 were purchased from Sigma-Aldrich (Darmstadt, Germany) and Tokyo Chemical Industry Co., Ltd. (Tokyo, Japan), respectively. Other reagents and solvents were available commercially and used without further purification.

### 3.2. Preparation of ORHB

A typical experimental procedure for the preparation of ORHB is as follows. A mixture of the commercially available PRHB (0.54 g) with sodium hydroxide (0.89 g) and chloroform (80 mL) was ultrasonicated at 50 °C for 1 h and then stirred at room temperature for 7 h. The reaction mixture was washed successively with 1 mol/L hydrochloric acid (5.0 mL) and water (80 mL). The chloroform layer was dried over anhydrous sodium sulfate, filtered, evaporated, and dried under reduced pressure to give ORHB (0.16 g). ^1^H-NMR (CDCl_3_): δ 1.22–1.27 (m, CH_3_ of main-chain), 1.85–1.88 (m, CH_3_ of terminal crotonate), 2.42–2.68 (m, CH_2_ of main-chain), 4.11–4.18 (m, CH of terminal 3-hydroxybutyrate), 5.18–5.36 (m, CH of main-chain), 5.76–5.87, 6.91–7.03 (m, =CH of terminal crotonate) (Figure 1). From the integrated ratio of the terminal methine signals at δ 4.11–4.18 and 5.76–5.87 to the main-chain methine signals at δ 5.18–5.36, the MW value was calculated to be ~530.

### 3.3. Preparation of Amylose–ORHB Inclusion Complex Using G_3_ as Primer

A mixture of G-1-P (0.204 g, 0.786 mmol), G_3_ (5.5 mg, 0.011 mmol), and ORHB (MW = 530, 0.159 g) in sodium acetate buffer (pH = 6.2, 0.2 mol/L, 5.0 mL) was ultrasonicated for 3 h to obtain a dispersion. After the addition of thermostable GP (14.4 U), the mixture was stirred at 70 °C for 48 h. The reaction mixture was then cooled to 45 °C over 7 h with stirring. The water-insoluble fraction was isolated by centrifugation, which was washed with water (25 mL), acetone (5.0 mL), and chloroform (30 mL), and dried under reduced pressure to give the inclusion complex (12.6 mg). ^1^H-NMR (DMSO-*d*_6_ + D_2_O) δ 1.19 (s, CH_3_ of ORHB), 2.53 (br, CH_2_ of ORHB, overlapping with DMSO), 3.31–3.92 (m, H_2_-H_6_ of amylose, overlapping with HOD), 4.98–5.18 (br, H_1_ of amylose, CH of ORHB).

### 3.4. Interaction between Single Amylose and ORHB in Thermostable Phosphorylase-Catalyzed Enzymatic Polymerization Using G_3_ as Primer

A mixture of G-1-P (0.207 g, 0.796 mmol), G_3_ (5.6 mg, 0.011 mmol), and ORHB (MW = 550, 0.106 g) in sodium acetate buffer (pH = 6.2, 0.2 mol/L, 4.0 mL) was ultrasonicated for 3 h to obtain a dispersion. After the addition of thermostable GP (14.4 U), the mixture was stirred at 70 °C for 48 h. The water-insoluble fraction was isolated by centrifugation, which was washed with water (30 mL) and chloroform (30 mL), and dried under reduced pressure to give the product (2.8 mg).

### 3.5. Preparation of Amylose–ORHB Inclusion Complex Using Water-Soluble Amylose as Primer

A mixture of G-1-P (0.129 g, 0.497 mmol), water-soluble amylose (MW = 2800, 13.9 mg, 5.0 μmol), and ORHB (MW = 530, 0.108 g) in sodium acetate buffer (pH = 6.2, 0.2 mol/L, 5.0 mL) was ultrasonicated for 3 h to obtain a dispersion. After the addition of thermostable GP (7.2 U), the mixture was stirred at 70 °C for 48 h. The reaction mixture was then cooled to 45 °C over 7 h with stirring. The water-insoluble fraction was isolated by centrifugation, which was washed with water (25 mL), acetone (5.0 mL), and chloroform (30 mL), and dried under reduced pressure to give the inclusion complex (23.4 mg). ^1^H-NMR (DMSO-*d*_6_ + D_2_O) δ 1.26 (s, CH_3_ of ORHB), 2.64 (br, CH_2_ of ORHB, overlapping with DMSO), 3.37–4.18 (m, H_2_-H_6_ of amylose, overlapping with HOD), 4.99–5.29 (br, H_1_ of amylose, CH of ORHB).

### 3.6. Measurements

Powder XRD measurements were conducted using a Rigaku Geigerflex RADIIB diffractometer (PANalytical B.V., EA Almelo, the Netherlands) with Ni-filtered CuKα radiation (*λ* = 0.15418 nm). ^1^H-NMR spectra were recorded on JEOL ECA 600 and ECX 400 spectrometers (JEOL, Akishima, Tokyo, Japan). IR spectra were recorded on a PerkinElmer Spectrum Two spectrometer (PerkinElmer Japan Co., Ltd., Yokohama, Japan).

## 4. Conclusions

When the thermostable GP-catalyzed enzymatic polymerization of G-1-P from the G_3_ primer was carried out in the presence of ORHB at 70 °C for 48 h and the mixture was subsequently cooled to 45 °C over 7 h, the amylose–ORHB inclusion complex was produced by vine-twining polymerization. On the other hand, the same system using the water-soluble amylose primer induced partial formation of the inclusion complex. The interaction between the single amylose obtained by the thermostable GP-catalyzed enzymatic polymerization at an elevated temperature and ORHB was speculated to be key for the formation of the inclusion complex in this study. As the two components in the inclusion complex are biodegradable polymers, we are going to investigate its biodegradability and biocompatibility. In the future, other biosynthesized polyesters, a family of PRHB, will be employed as guest polymers in the present vine-twining polymerization system to produce the corresponding inclusion complexes with different structures.

## Data Availability

Not applicable.
